# Supplementary material for: Phacoemulsification Induced Changes of Choroidal Thickness in Eyes with Age-Related Macular Degeneration
Source: Medicina (Kaunas). 2020 May 22;56(5):252. doi: 10.3390/medicina56050252 (PMC7279142; doi:10.3390/medicina56050252)
Supplement: Supplementary file 1 [file medicina-56-00252-s001.pdf]

**Table S1.** Univariate linear regression analysis of different ocular factors affecting the CT changes in both groups.

| | **AMD group** | | | | | | | | **No-AMD group** | | | | | | | |
|---|---|---|---|---|---|---|---|---|---|---|---|---|---|---|---|---|
| | **Age** | | **Sex** | | **AL** | | **P IOP** | | **Age** | | **Sex** | | **AL** | | **P IOP** | |
| **Area** | β | p | β | p | β | p | β | p | β | p | β | p | β | p | β | p |
| *C* | | | | | | | | | | | | | | | | |
| **P** | -3.4 | 0.12 | 47.7 | 0.13 | -45.5 | 0.00 | 4.6 | 0.45 | -3.62 | 0.02 | 16.1 | 0.58 | -21.9 | 0.15 | -3.49 | 0.55 |
| **M1** | -3.1 | 0.12 | 23.5 | 0.41 | -37.7 | 0.00 | 4.2 | 0.45 | -4.63 | 0.01 | 19.8 | 0.51 | -29.3 | 0.05 | -2.96 | 0.62 |
| **M3** | -5.5 | 0.03 | 28.2 | 0.40 | -43.2 | 0.00 | 7.6 | 0.22 | -5.87 | 0.00 | 9.0 | 0.75 | -25.7 | 0.06 | 2.05 | 0.72 |
| iNAS | | | | | | | | | | | | | | | | |
| **P** | -3.3 | 0.12 | 50.9 | 0.08 | -42.4 | 0.00 | 4.0 | 0.49 | -3.9 | 0.01 | 3.9 | 0.89 | -26.3 | 0.07 | -2.2 | 0.71 |
| **M1** | 3.7 | 0.04 | 30.6 | 0.26 | -33.0 | 0.00 | 4.4 | 0.41 | -4.5 | 0.01 | -1.5 | 0.96 | -28.7 | 0.04 | -2.4 | 0.68 |
| **M3** | -4.5 | 0.08 | 38.3 | 0.24 | -40.9 | 0.00 | 6.1 | 0.33 | -6.0 | 0.00 | 0.9 | 0.98 | -27.7 | 0.07 | 0.6 | 0.93 |
| *oNAS* | | | | | | | | | | | | | | | | |
| **P** | -2.7 | 0.12 | 22.1 | 0.36 | -32.8 | 0.00 | 5.7 | 0.22 | -3.7 | 0.01 | -0.3 | 0.99 | -22.9 | 0.07 | -0.4 | 0.81 |
| **M1** | -1.9 | 0.27 | 33.6 | 0.19 | -34.2 | 0.00 | 4.9 | 0.33 | -3.3 | 0.02 | -13.1 | 0.61 | -21.1 | 0.11 | 29.2 | 0.66 |
| **M3** | -2.9 | 0.16 | 27.8 | 0.32 | -36.5 | 0.00 | 5.9 | 0.25 | -3.7 | 0.01 | 4.2 | 0.88 | -25.9 | 0.05 | 1.3 | 0.82 |
| *iTEM* | | | | | | | | | | | | | | | | |
| **P** | -2.7 | 0.21 | 52.3 | 0.99 | -39.1 | 0.00 | 3.1 | 0.60 | -3.7 | 0.01 | 26.8 | 0.25 | -23.3 | 0.05 | -1.4 | 0.77 |
| **M1** | -3.3 | 0.08 | 30.6 | 0.26 | -3.0 | 0.01 | 3.1 | 0.57 | -4.3 | 0.00 | 21.1 | 0.38 | -25.0 | 0.03 | 10.1 | 0.87 |
| **M3** | -4.5 | 0.09 | 29.8 | 0.39 | -38.8 | 0.00 | 8.2 | 0.19 | -5.4 | 0.00 | 17.4 | 0.49 | -26.0 | 0.03 | 1.4 | 0.79 |
| *oTEM* | | | | | | | | | | | | | | | | |
| **P** | -2.3 | 0.23 | 39.6 | 0.14 | -29.0 | 0.02 | 2.2 | 0.67 | -3.9 | 0.00 | 20.1 | 0.32 | -18.2 | 0.07 | 1.3 | 0.75 |
| **M1** | -2.9 | 0.09 | 28.9 | 0.26 | -24.9 | 0.03 | 1.1 | 0.83 | -4.1 | 0.00 | 6.0 | 0.78 | -17.5 | 0.09 | 0.6 | 0.89 |
| **M3** | -4.4 | 0.04 | 30.5 | 0.29 | -31.9 | 0.01 | 4.3 | 0.42 | -5.5 | 0.00 | 4.0 | 0.86 | -11.3 | 0.31 | -1.3 | 0.84 |
| *iSUP* | | | | | | | | | | | | | | | | |
| **P** | -3.1 | 0.15 | 55.8 | 0.05 | -34.7 | 0.01 | 4.1 | 0.48 | -4.3 | 0.01 | 19.4 | 0.51 | -32.1 | 0.03 | -1.1 | 0.86 |
| **M1** | -2.7 | 0.19 | 56.7 | 0.05 | -32.1 | 0.02 | 4.1 | 0.48 | -3.0 | 0.09 | -8.9 | 0.79 | -31.1 | 0.07 | -3.2 | 0.64 |
| **M3** | -4.9 | 0.07 | 35.9 | 0.30 | -29.2 | 0.04 | 7.4 | 0.25 | -5.9 | 0.00 | 20.2 | 0.52 | -30.8 | 0.04 | -1.3 | 0.84 |
| *oSUP* | | | | | | | | | | | | | | | | |
| **P** | -3.1 | 0.10 | 46.7 | 0.08 | -29.6 | 0.01 | 2.4 | 0.64 | -4.1 | 0.02 | 2.7 | 0.39 | -23.5 | 0.15 | -2.2 | 0.73 |
| **M1** | -3.3 | 0.04 | 45.4 | 0.05 | -22.9 | 0.03 | 0.9 | 0.85 | -3.6 | 0.05 | -3.2 | 0.92 | -20.3 | 0.25 | -4.3 | 0.04 |
| **M3** | -3.6 | 0.11 | 43.1 | 0.13 | -24.5 | 0.04 | 2.5 | 0.64 | -5.6 | 0.02 | 16.8 | 0.58 | -21.4 | 0.17 | 1.1 | 0.87 |
| *iINF* | | | | | | | | | | | | | | | | |
| **P** | -3.8 | 0.09 | 29.7 | 0.36 | -46.1 | 0.00 | 4.5 | 0.46 | -3.8 | 0.01 | -4.0 | 0.88 | -26.9 | 0.03 | 3.2 | 0.53 |
| **M1** | -3.2 | 0.08 | 12.3 | 0.66 | -41.1 | 0.00 | 4.8 | 0.38 | -4.4 | 0.00 | -10.7 | 0.67 | -22.4 | 0.08 | 2.6 | 0.61 |
| **M3** | -5.8 | 0.02 | 15.2 | 0.65 | -42.5 | 0.00 | 9.5 | 0.11 | -5.6 | 0.02 | 16.8 | 0.58 | -21.4 | 0.17 | 3.7 | 0.51 |
| *oINF* | | | | | | | | | | | | | | | | |
| **P** | -3.3 | 0.05 | 5.5 | 0.83 | -36.6 | 0.00 | 4.2 | 0.37 | -3.2 | 0.01 | -12.6 | 0.58 | -15.7 | 0.17 | 4.0 | 0.38 |
| **M1** | -3.5 | 0.03 | 4.7 | 0.84 | -36.9 | 0.00 | 4.5 | 0.34 | -3.8 | 0.00 | -13.7 | 0.54 | -14.9 | 0.19 | 3.6 | 0.43 |
| **M3** | -3.9 | 0.08 | -19.9 | 0.51 | -36.5 | 0.00 | 6.7 | 0.23 | -4.2 | 0.00 | -9.8 | 0.69 | -15.8 | 0.20 | 4.2 | 0.39 |

C - central ring; iNAS - nasal inner; iSUP - superior inner; iTEM - temporal inner; iINF - inferior inner; oNAS - nasal outer; oSUP - superior outer; oTEM - temporal outer; oINF - inferior outer regions.

β - regression coefficient; P IOP - preoperative intraocular pressure; p - p-value; $R^2$ - coefficient of determination.
